# ONO-AE3-208 inhibits myeloid derived suppressor cells and glioma growth

**DOI:** 10.1186/2051-1426-2-S3-P217

**Published:** 2014-11-06

**Authors:** Gary Kohanbash, Erin Straw, Brian Ahn, Hideho Okada

**Affiliations:** 1 University of California San Francisco, San Francisco, CA, USA; 2 University of Pittsburgh, Pittsburgh, PA, USA

## 

Myeloid Derived Suppressor Cells (MDSCs) heavily infiltrate in a variety of solid tumors and suppress anti-tumor T-cell activity. Our recent studies have demonstrated the ability of monocytic, Ly6C+ MDSCs to promote glioma growth through the activation of cyclooxygenase (COX)-2 pathway, which is responsible for prostaglandin-synthesis. ONO-AE3-208 is an antagonist of the prostaglandin E (EP)-4 receptor, which is an important positive feedback regulator of the COX-2 pathway. We thus examined the ability of ONO-AE3-208 to suppress MDSC activity in gliomas. ONO-AE3-208 treatment in mice bearing established GL261-quad glioma in the brain resulted in complete and persistent rejection of the tumors. Flow cytometric analysis revealed that gliomas in the ONO-AE3-208-treated mice were infiltrated by fewer numbers of Ly6C+ MDSCs compared with non-treated animals. We subsequently isolated glioma-infiltrating Ly6C+ MDSCs by flow-sorting to address their functions. RT-PCR analysis revealed that the Ly6C+ MDSCs derived from ONO-AE3-208 treated mice expressed lower levels of the Arg1 and Cox2 expression compared to control animals. Consistently, brain infiltrating leukocytes in ONO-AE3-208 treated tumor-bearing mice demonstrated enhanced IFN-g expression compared with control mice, suggestive of enhanced T-cell activity. Importantly, ONO-AE3-208 inhibited glioma growth and promoted immune activity in 2 additional murine glioma models: the Sleeping Beauty de novo glioma model and the SB28 glioma cell line model. Our data demonstrate that ONO-AE3-208 may be useful in the treatment of glioma patients to suppress Ly6C+ MDSCs and promote anti-tumor immunity.

